# Elevated neutrophil to high-density lipoprotein ratio predicts pneumonia in patients with intracerebral hemorrhage

**DOI:** 10.3389/fmed.2025.1572131

**Published:** 2025-07-29

**Authors:** Yuning Feng, Wenyao Cui, Yangchun Xiao, Xin Cheng, Lvlin Chen, Liyuan Peng, Chao You, Fang Fang, Li Li, Dianxiang Lu, Yu Zhang

**Affiliations:** ^1^Affiliated Hospital and Clinical Medical College of Chengdu University, Chengdu, China; ^2^West China Hospital, Sichuan University, Chengdum, China; ^3^First People's Hospital of Longquanyi District, Chengdu, China

**Keywords:** neutrophil-to-high-density lipoprotein cholesterol ratio, pneumonia, intracerebral hemorrhage, biomarker, inflammation

## Abstract

**Background:**

Pneumonia is a common and serious complication among patients with intracerebral hemorrhage (ICH), contributing to increased morbidity and mortality. Early identification of patients at high risk for pneumonia is essential for implementing preventive strategies and improving outcomes. The neutrophil-to-high-density lipoprotein cholesterol ratio (NHR) has emerged as a novel marker of systemic inflammation with potential predictive value. This study examines whether elevated NHR is associated with an increased risk of pneumonia in patients with ICH, aiming to evaluate NHR as a practical biomarker for early risk prediction.

**Methods:**

This retrospective multicenter cohort study included patients diagnosed with intracerebral hemorrhage (ICH) from three hospitals: West China Hospital of Sichuan University (December 2010 to August 2019), The First People’s Hospital of Longquanyi District, Chengdu (December 2016 to November 2020), and the Affiliated Hospital of Chengdu University (August 2012 to November 2020). Patients were stratified into two groups based on their NHR: less than 6.84 and 6.84 or greater. The primary outcome was the occurrence of pneumonia during hospitalization.

**Results:**

Among the 2,639 patients, the incidence of pneumonia was 27.5% (363 of 1,320) in the low NHR group (NHR < 6.84) and 47.4% (625 of 1,319) in the high NHR group (NHR ≥ 6.84). Unadjusted models showed that patients in the high NHR group had a significantly increased risk of developing pneumonia compared to those in the low NHR group (odds ratio [OR], 2.37; 95% confidence interval [CI], 2.02–2.79; *p* < 0.001). After adjusting for confounding factors, this association remained significant (adjusted OR, 2.64; 95% CI, 1.37–5.07; *p* = 0.004). Furthermore, when analyzing NHR as a continuous variable, each unit increase in NHR was associated with an increased risk of pneumonia (adjusted OR, 1.03; 95% CI, 1.01–1.04; *p* < 0.001).

**Conclusion:**

Elevated NHR was significantly associated with an increased risk of pneumonia among patients with intracerebral hemorrhage. Given its simplicity and potential diagnostic value, NHR may serve as a useful biomarker for predicting pneumonia risk in this population, facilitating early identification and potentially improving patient outcomes.

## Introduction

Intracerebral hemorrhage (ICH) accounts for approximately 10 to 15% of all strokes and is associated with high morbidity and mortality globally (1, 2). Despite advances in medical care, patients with ICH remain at substantial risk for complications, among which pneumonia is the most common, occurring in up to 30 to 60% of cases (3, 4). Pneumonia significantly worsens clinical outcomes in these patients, leading to prolonged hospitalization, increased healthcare costs, and a more than twofold increase in mortality (5, 6). Therefore, early prediction and prevention of pneumonia are critical for improving patient outcomes.

Current predictive models for pneumonia in patients with ICH are limited by complexity and lack of accuracy, making them impractical for routine clinical use (7). Traditional risk factors such as age, stroke severity, and comorbidities provide insufficient predictive power. Therefore, there is a need for simple and reliable biomarkers that can be readily measured upon hospital admission to identify patients at high risk of pneumonia.

The neutrophil-to-high-density lipoprotein cholesterol ratio (NHR) has recently emerged as a novel marker of systemic inflammation (8). Neutrophils are key players in the innate immune response and are elevated in various inflammatory conditions (9). In contrast, high-density lipoprotein cholesterol (HDL-C) possesses anti-inflammatory and antioxidant properties; lower levels are associated with an increased risk of infections (10). The NHR combines these two parameters, potentially providing a more sensitive indicator of inflammatory status than either parameter alone.

Previous studies have demonstrated that elevated NHR is associated with poor outcomes in cardiovascular diseases and certain cancers (11, 12). However, the role of NHR in predicting pneumonia risk among patients with ICH has not been explored. We hypothesized that elevated NHR on admission would predict the risk of pneumonia in patients with ICH. This study aimed to evaluate the association between NHR and the incidence of pneumonia in patients with ICH, assessing its potential as a practical biomarker for early risk stratification.

## Methods

### Study design

This retrospective, multicenter cohort study included 2,639 patients diagnosed with ICH from three medical institutions: West China Hospital of Sichuan University (December 2010 to August 2019), The First People’s Hospital of Longquanyi District, Chengdu (December 2016 to November 2020), and the Affiliated Hospital of Chengdu University (August 2012 to November 2020). Ethical approval for the study was obtained from the institutional review boards of all participating hospitals: West China Hospital (Approval No. 2021-624), The First People’s Hospital of Longquanyi District, Chengdu (Approval No. AF-AK86 2,022,010), and the Affiliated Hospital of Chengdu University (Approval No. PJ2021-017-03). Given the study’s retrospective design and its classification as a clinical audit, the requirement for informed consent was waived. This study was conducted in accordance with the STROBE reporting guidelines and adhered to the ethical principles outlined in the Declaration of Helsinki (1964). The diagnoses of ICH and pneumonia were standardized across all three centers, following uniform protocols and guidelines to ensure consistency.

### Patient selection

This study included all patients diagnosed with spontaneous ICH. Diagnosis was confirmed at the time of admission through computed tomography (CT) or magnetic resonance imaging (MRI), and, where applicable, further verified via intraoperative assessment by a neurosurgeon during hospitalization. Patients aged 18 years or older were eligible for inclusion. Exclusion criteria were as follows: (1) ischemic stroke with hemorrhagic transformation, traumatic brain injury, cerebral aneurysm, arteriovenous malformations, coagulopathy-related hemorrhagic disorders, or any other condition not classified as primary ICH; (2) pre-existing diagnosis of pneumonia prior to admission; and (3) Absence of serum NHR measurement within not 24 h of admission.

### Data collection

Demographic and clinical data were collected, including age, sex, smoking history, alcohol use, hypertension, diabetes mellitus, hematoma location, hematoma volume, and the presence of intraventricular hemorrhage. The severity of ICH at admission was assessed using the Glasgow Coma Scale (GCS), and detailed treatment information was recorded. Comprehensive laboratory tests, including NHR, leukocyte count, lymphocyte count, and platelet levels, were conducted within 24 h of admission. Receiver operating characteristic (ROC) curve analysis was utilized to determine the optimal discriminatory value of NHR, with the cutoff point established based on the Youden index. The primary outcome of interest was the incidence of pneumonia. The statistical analysis of this study was conducted by a statistician, ensuring the selection of appropriate data analysis methods and the scientific interpretation of the results.

### Definition of pneumonia

Pneumonia was diagnosed based on clinical, laboratory, and radiological findings, following established guidelines. Patients were considered to have pneumonia if they exhibited new or progressive infiltrates on chest radiographs along with at least two of the following criteria: (1) fever (>38°C) or hypothermia (<36°C); (2) leukocytosis (white blood cell count >10 × 10^9^/L) or leukopenia (<4 × 10^9^/L); (3) purulent sputum or increased respiratory secretions; (4) worsening cough or dyspnea; and (5) positive sputum culture for pathogenic organisms. The diagnosis was confirmed by the attending physician or a pulmonologist (13).

### Statistical analysis

All statistical analyses were performed using R version 4.2.1. Continuous variables are reported as means with standard deviations, while categorical variables are presented as frequencies with proportions. Differences in medians and proportions between the two NHR groups were assessed using the Kruskal-Wallis test, Fisher’s exact test, or Chi-squared test, as appropriate. Missing data were addressed using multiple imputation methods. The association between NHR and pneumonia was examined using restricted cubic splines to explore the relationship’s pattern and magnitude. Adjusted multivariate logistic regression models were constructed to assess the relationships between perioperative variables and outcomes, with results presented as odds ratios (OR) and 95% confidence intervals (CI). Variables with a *p* value less than 0.10 in univariate analyses or those with demonstrated relevance in previous research were included in the multivariate models. Additionally, subgroup analyses were conducted to identify factors associated with pneumonia within each NHR group.

## Results

The study comprised 2,639 patients diagnosed with ICH from three institutions: West China Hospital of Sichuan University, The First People’s Hospital of Longquanyi District, Chengdu, and The Affiliated Hospital of Chengdu University, all of whom were aged 18 years or older. Patients with conditions other than primary ICH and those lacking NHR measurements at admission were excluded (Table 1). Stratified analyses by center revealed no significant differences in the association between NHR and pneumonia risk (P for interaction > 0.05). Therefore, data from all centers were pooled for the analyses.

**Table 1:** Baseline characteristics of the patients.

Characteristics	NHR			*p*	Missvalue (*n*.%)
	Overall	<6.84	>6.84		
	(*n* = 2,639)	(*n* = 1,320)	(*n* = 1,319)		
Demographics					
Age (mean (SD))	56.46 (14.54)	58.36 (14.73)	54.56 (14.09)	<0.001	0
Female (%)	836 (31.7)	478 (36.2)	358 (27.1)	<0.001	0
Alcohol, *n* (%)	826 (31.3)	398 (30.2)	428 (32.4)	0.218	0
Smoking, *n* (%)					
Never	1805 (68.4)	918 (69.5)	887 (67.2)		
Current	689 (26.1)	321 (24.3)	368 (27.9)		
Ever	145 (5.5)	81 (6.1)	64 (4.9)		
Medical history, *n* (%)					
Hypertension	1928 (73.1)	930 (70.5)	998 (75.7)	0.003	0
Diabetes	271 (10.3)	122 (9.2)	149 (11.3)	0.094	0
Hematoma characteristics					
Hematoma intraventricular	642 (24.3)	290 (22.0)	352 (26.7)	0.005	0
Hematoma size (mean (SD))	23.97 (27.82)	19.56 (25.40)	28.57 (29.46)	<0.001	261 (9.9)
Hematoma infratentorial = Infratentorial	561 (21.3)	255 (19.3)	306 (23.2)	0.007	0
Laboratory tests, mean (SD)					
Neutrophil count (mean (SD))	9.20 (5.38)	6.57 (2.48)	11.84 (6.16)	<0.001	0
High-density lipoprotein (mean (SD))	1.29 (0.47)	1.50 (0.45)	1.07 (0.38)	<0.001	0
GCS (mean (SD))	10.50 (4.20)	11.78 (3.78)	9.22 (4.21)	<0.001	0

GCS: Glasgow Coma Scale.

Values represent the number of patients (%) unless indicated otherwise.

*p* values for trend for continuous variables are from a generalized linear model, and those for categorical variables are from an ordinal or logistic regression.

In our study, we performed subgroup analyses to assess whether the association between the neutrophil-to-high-density lipoprotein cholesterol ratio (NHR) and pneumonia risk was consistent across different patient characteristics. The subgroups were based on age (<60 vs. ≥60 years), sex (male vs. female), hypertension status (presence vs. absence), presence of intraventricular hemorrhage, Glasgow Coma Scale (GCS) scores (≤8 vs. >8) and so on. The results demonstrated that elevated NHR was consistently associated with an increased risk of pneumonia across all subgroups, with no significant interactions detected (all P for interaction > 0.05), indicating that the predictive value of NHR is robust regardless of these factors. Additionally, we utilized a restricted cubic spline regression model to explore the dose–response relationship between NHR and pneumonia risk. The spline curve revealed a positive linear association, suggesting that as NHR increases, the risk of pneumonia also increases proportionally. This finding further supports the role of NHR as a continuous predictive biomarker for pneumonia in patients with intracerebral hemorrhage.

The ROC curve analysis showed that the area under the curve (AUC) for NHR in predicting pneumonia was 0.70 (95% confidence interval, 0.68–0.72; *p* < 0.001). Based on the Youden Index, the optimal cutoff value for NHR was determined to be 6.84, which provided a sensitivity of 63% and a specificity of 65%. Based on this threshold, patients were divided into two groups: a lower NHR group (≤6.84) and a higher NHR group (>6.84). The higher NHR group comprised 1,319 patients, while the lower NHR group included 1,320 patients. Table 1 provides a comparison of patient demographics, medical history, and other relevant factors between the two groups. Higher NHR values were correlated with age, sex, hypertension, intraventricular hemorrhage, GCS score, glucose levels, C-reactive protein, absolute lymphocyte count, albumin levels, and absolute monocyte count.

NHR levels were measured within 24 h of admission for all patients. Pneumonia was diagnosed at a median of 5 days post-admission (IQR, 3–7 days). Notably, 85% of pneumonia cases occurred after NHR measurement, confirming that elevated NHR values were present before the onset of pneumonia. This finding supports the utility of admission NHR levels as a predictive marker for subsequent pneumonia development in ICH patients.

Overall, among the 2,639 patients, pneumonia occurred in 27.5% (363 of 1,320) of the low NHR group (NHR < 6.84) and 47.4% (625 of 1,319) of the high NHR group (NHR ≥ 6.84). Unadjusted models showed that patients in the high NHR group had a significantly increased risk of pneumonia compared to those in the low NHR group (odds ratio [OR], 2.37; 95% confidence interval [CI], 2.02–2.79; *p* < 0.001). After adjusting for confounding factors, this association remained significant (adjusted OR, 2.64; 95% CI, 1.37–5.07; *p* = 0.004). Additionally, analyzing NHR as a continuous variable revealed that each unit increase in NHR was associated with an increased risk of pneumonia (adjusted OR, 1.03; 95% CI, 1.01–1.04; *p* < 0.001) (Table 2).

**Table 2:** Unadjusted and adjusted associations between NHR and pneumonia.

	NHR (%)	Events, *n* (%)	Unadjusted		Multivariable regression adjusted	
			OR (95% CI)	*p*	OR (95% C1)	*p*
Continuous	per 1	NA	1.05 (1.04–1.07)	<0.001	1.03 (1.01–1.04)	<0.001
Dichotomy	<6.84	363/1320 (27.5%)	1(reference)		1(reference)	
	>6.84	625/1319 (47.4%)	2.37 (2.02–2.79)	<0.001	2.64 (1.37–5.07)	0.004
Quartile						
Q1	0.49–4.61	155/660 (23.5%)	1(reference)		1(reference)	
Q2	4.61–6.84	208/660 (31.5%)	1.50 (1.18–1.91)	0.001	1.22 (0.94–1.60)	0.142
Q3	6.84–9.96	290/659 (44%)	2.56 (2.02–3.24)	<0.001	1.88 (1.44–2.45)	<0.001
Q4	>9.96	335/660 (50.8%)	3.36 (2.65–4.25)	<0.001	1.75 (1.31–2.34)	<0.001

Cl, confidence interval; NA, not available; OR, odds ratio.

Multivariable regression adjusted including age, sex, hypertension, hematoma intraventricular, GCS, glucose, C reactive protein, lymphocyte absolute value, albumin, and monocyte absolute value.

## Discussion

In this large cohort study of patients with ICH, we found that a higher NHR on admission was independently associated with an increased risk of pneumonia. The overall incidence of pneumonia was 37.4%. Patients in the higher NHR group had a significantly higher pneumonia rate compared with those in the lower NHR group (47.4% vs. 27.5%; *p* < 0.001). In both unadjusted and adjusted analyses, each unit increase in NHR was associated with an increased odds of developing pneumonia (unadjusted odds ratio [OR], 1.05; 95% CI, 1.04–1.07; adjusted OR, 1.03; 95% CI, 1.01–1.04; *p* < 0.001).

The biological plausibility of NHR as a predictor of pneumonia lies in its reflection of systemic inflammatory and immune status. Neutrophils are key players in the innate immune response and are elevated in various inflammatory conditions, including acute stroke (14). An increased neutrophil count may indicate an exaggerated inflammatory response, which can compromise the immune system and predispose patients to infections (15). Conversely, high-density lipoprotein cholesterol (HDL-C) possesses anti-inflammatory and antioxidant properties (16). Lower levels of HDL-C have been associated with impaired immune function and increased susceptibility to infections (17). The NHR combines these two parameters, potentially serving as a sensitive marker of the balance between proinflammatory and anti-inflammatory states.

Our findings suggest that NHR is an independent predictor of pneumonia among patients with ICH. The persistence of the association after adjusting for potential confounders indicates that NHR provides additional predictive value beyond traditional risk factors such as age, stroke severity, and comorbidities. To our knowledge, this is the first study to demonstrate the utility of NHR as a biomarker for predicting pneumonia risk in this patient population. Previous studies have identified elevated NHR as a prognostic marker in cardiovascular diseases and certain infections, such as sepsis. Our results extend these findings to patients with ICH, highlighting the potential role of NHR in identifying those at higher risk for pneumonia. This aligns with research indicating that systemic inflammation plays a pivotal role in post-stroke complications, including infections.

Current clinical guidelines for the management of patients with ICH emphasize the importance of preventing infections, including pneumonia, through strategies such as early mobilization, swallowing assessments, and vigilant monitoring of respiratory status (18). However, these guidelines primarily focus on clinical risk factors and standard preventive measures without incorporating specific biomarkers for risk stratification. Our findings suggest that NHR could serve as an additional tool to enhance existing guidelines by providing an objective, easily measurable parameter to identify patients at higher risk for pneumonia. Integrating NHR into clinical protocols may allow for more targeted preventive interventions, optimizing resource allocation and potentially improving patient outcomes.

The clinical implications of our study are significant. Pneumonia is a common and serious complication after ICH, associated with prolonged hospitalization, increased healthcare costs, higher disability rates, and increased mortality. Early identification of patients at high risk for pneumonia allows for the implementation of preventive strategies. NHR is a simple, cost-effective, and readily available laboratory parameter that can be measured upon hospital admission. Incorporating NHR into clinical practice could enhance risk stratification models and guide clinical decision-making. Future studies should explore the integration of NHR with other clinical and laboratory parameters to develop comprehensive risk prediction tools.

While our study establishes a significant association between elevated NHR and increased pneumonia risk in patients with intracerebral hemorrhage, further research is warranted to enhance these findings. Future studies should focus on conducting prospective, multicenter trials to determine a causal relationship between NHR levels and pneumonia development. Such studies would help to confirm our results and address potential biases inherent in retrospective designs. Additionally, exploring the combined application of NHR with other inflammatory biomarkers—such as C-reactive protein, procalcitonin, or the neutrophil-to-lymphocyte ratio—may improve predictive accuracy and provide a more comprehensive assessment of a patient’s inflammatory status. Investigating the integration of NHR into existing risk stratification models could also facilitate the development of personalized prevention strategies, ultimately improving clinical outcomes for patients with intracerebral hemorrhage.

This study has several limitations. First, as an observational study, causality cannot be established between elevated NHR and pneumonia risk. Second, we did not assess other inflammatory markers, such as C-reactive protein or procalcitonin, which might provide additional predictive value. Moreover, we were unable to directly compare the predictive performance of NHR with other established inflammatory biomarkers, such as the neutrophil-to-lymphocyte ratio (NLR), platelet-to-lymphocyte ratio (PLR), or monocyte-to-lymphocyte ratio (MLR). This limitation was due to the retrospective, multicenter design and reliance on routine clinical data collection, resulting in substantial missing laboratory data and timing inconsistencies. Specifically, lymphocyte, platelet, and monocyte counts were not consistently measured within the first 24 h of admission, making reliable comparisons impossible. Future prospective studies with standardized protocols are essential to comprehensively compare these inflammatory markers.

Third, due to the retrospective design of our study, there is a potential for selection bias, and the results may have limited generalizability to other populations. Specifically, the exclusion of patients who lacked NHR measurements within 24 h of admission could have introduced selection bias, potentially affecting the representativeness and applicability of our findings. Additionally, although we identified an optimal NHR cutoff value (6.84) using ROC curve analysis based on the Youden index, this value has not been externally validated across diverse patient populations. Future multicenter, prospective validation studies are required to determine the generalizability and clinical applicability of this cutoff value.

## Conclusion

Elevated NHR was significantly associated with an increased risk of pneumonia among patients with intracerebral hemorrhage. Given its simplicity and potential diagnostic value, NHR may serve as a useful biomarker for predicting pneumonia risk in this population, facilitating early identification and potentially improving patient outcomes.

## Data Availability

The raw data supporting the conclusions of this article will be made available by the authors, without undue reservation.

## References

[1] Rajashekar D Liang JW. Intracerebral hemorrhage. Treasure Island (FL): StatPearls (2024). 31971743

[2] Gil-Garcia CA Flores-Alvarez E Cebrian-Garcia R Mendoza-Lopez AC Gonzalez-Hermosillo LM Garcia-Blanco MDC. Essential topics about the imaging diagnosis and treatment of hemorrhagic stroke: a comprehensive review of the 2022 AHA guidelines. Curr Probl Cardiol. (2022) 47:101328. doi: 10.1016/j.cpcardiol.2022.101328, PMID: 35870549

[3] Hostettler IC Seiffge DJ Werring DJ. Intracerebral hemorrhage: an update on diagnosis and treatment. Expert Rev Neurother. (2019) 19:679–94. doi: 10.1080/14737175.2019.1623671, PMID: 31188036

[4] Jones HR Robb CT Perretti M Rossi AG. The role of neutrophils in inflammation resolution. Semin Immunol. (2016) 28:137–45. doi: 10.1016/j.smim.2016.03.007, PMID: 27021499

[5] de Oliveira S Rosowski EE Huttenlocher A. Neutrophil migration in infection and wound repair: going forward in reverse. Nat Rev Immunol. (2016) 16:378–91. doi: 10.1038/nri.2016.49, PMID: 27231052 PMC5367630

[6] van der Linden M Meyaard L. Fine-tuning neutrophil activation: strategies and consequences. Immunol Lett. (2016) 178:3–9. doi: 10.1016/j.imlet.2016.05.015, PMID: 27262927

[7] Li F Weng G Zhou H Zhang W Deng B Luo Y. The neutrophil-to-lymphocyte ratio, lymphocyte-to-monocyte ratio, and neutrophil-to-high-density-lipoprotein ratio are correlated with the severity of Parkinson's disease. Front Neurol. (2024) 15:1322228. doi: 10.3389/fneur.2024.1322228, PMID: 38322584 PMC10844449

[8] Qing G Bao C Yang Y Wei B. Association between neutrophil to high-density lipoprotein cholesterol ratio (NHR) and depression symptoms among the United States adults: a cross-sectional study. Lipids Health Dis. (2024) 23:215. doi: 10.1186/s12944-024-02204-y, PMID: 39003458 PMC11245866

[9] Yu L Ma K Hao J Zhang B. Neutrophil to high-density lipoprotein cholesterol ratio, a novel risk factor associated with acute ischemic stroke. Medicine (Baltimore). (2023) 102:e34173. doi: 10.1097/MD.0000000000034173, PMID: 37390238 PMC10313256

[10] Zhang A Zhu Y Liao J Wu D Yan X Chen J. The Association of Systemic Inflammatory Response Index and Neutrophil-to-High-Density Lipoprotein Ratio Mediated by fasting blood glucose with 90-day prognosis in acute ischemic stroke patients. Neuroepidemiology. (2025) 59:1–12. doi: 10.1159/000539132, PMID: 38749405 PMC11797957

[11] Huang JB Chen YS Ji HY Xie WM Jiang J Ran LS. Neutrophil to high-density lipoprotein ratio has a superior prognostic value in elderly patients with acute myocardial infarction: a comparison study. Lipids Health Dis. (2020) 19:59. doi: 10.1186/s12944-020-01238-2, PMID: 32247314 PMC7126405

[12] Lamichhane P Agrawal A Abouainain Y Abousahle S Regmi PR. Utility of neutrophil-to-high-density lipoprotein-cholesterol ratio in patients with coronary artery disease: a narrative review. J Int Med Res. (2023) 51:3000605231166518. doi: 10.1177/03000605231166518, PMID: 37038922 PMC10107976

[13] National Institute for Health and Care Excellence (NICE). Pneumonia in adults: diagnosis and management. (Clinical Guideline CG191). London, UK: National Institute for Health and Care Excellence. (2023).

[14] Chinese Society of Neurosurgery of the Chinese Medical Association and Chinese Neurosurgical Critical Care Management Collaborative Group. Chinese expert consensus on the diagnosis and treatment of infections in neurosurgical critically ill patients (2017). Chin Med J. (2017) 97:1607–14. doi: 10.3760/cma.j.issn.0376-2491.2017.21.005

[15] Dyckhoff-Shen S Koedel U Brouwer MC Bodilsen J Klein M. ChatGPT fails challenging the recent ESCMID brain abscess guideline. J Neurol. (2024) 271:2086–101. doi: 10.1007/s00415-023-12168-1, PMID: 38279999 PMC10972965

[16] Lee M Lee SY Bae YS. Emerging roles of neutrophils in immune homeostasis. BMB Rep. (2022) 55:473–80. doi: 10.5483/BMBRep.2022.55.10.115, PMID: 36104260 PMC9623243

[17] De Nardo D Labzin LI Kono H Seki R Schmidt SV Beyer M. High-density lipoprotein mediates anti-inflammatory reprogramming of macrophages via the transcriptional regulator ATF3. Nat Immunol. (2014) 15:152–60. doi: 10.1038/ni.2784, PMID: 24317040 PMC4009731

[18] Stamp MEM Halwes M Nisbet D Collins DJ. Breaking barriers: exploring mechanisms behind opening the blood-brain barrier. Fluids Barriers CNS. (2023) 20:87. doi: 10.1186/s12987-023-00489-2, PMID: 38017530 PMC10683235

