# Supplementary figures and images for: Elevated neutrophil to high-density lipoprotein ratio predicts pneumonia in patients with intracerebral hemorrhage
Source: Front Med (Lausanne). 2025 Jul 29;12:1572131. doi: 10.3389/fmed.2025.1572131 (PMC12340996; doi:10.3389/fmed.2025.1572131)

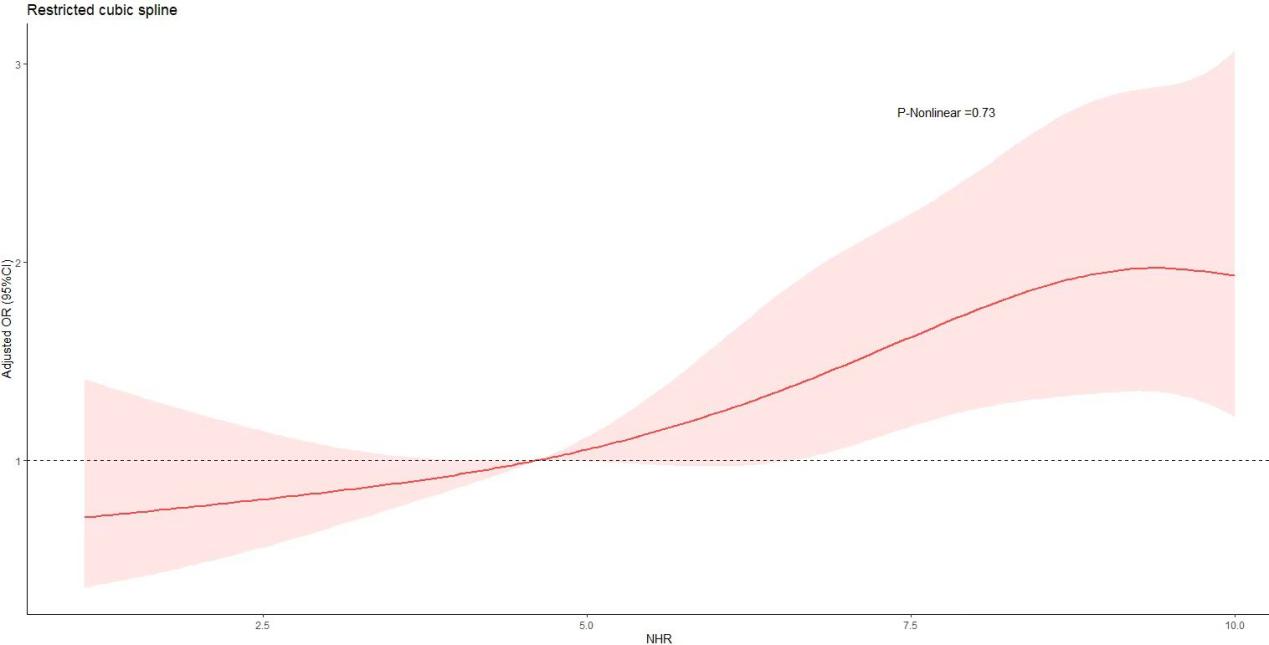

Supplement: Supplementary file 1 [file Image_1.jpeg]

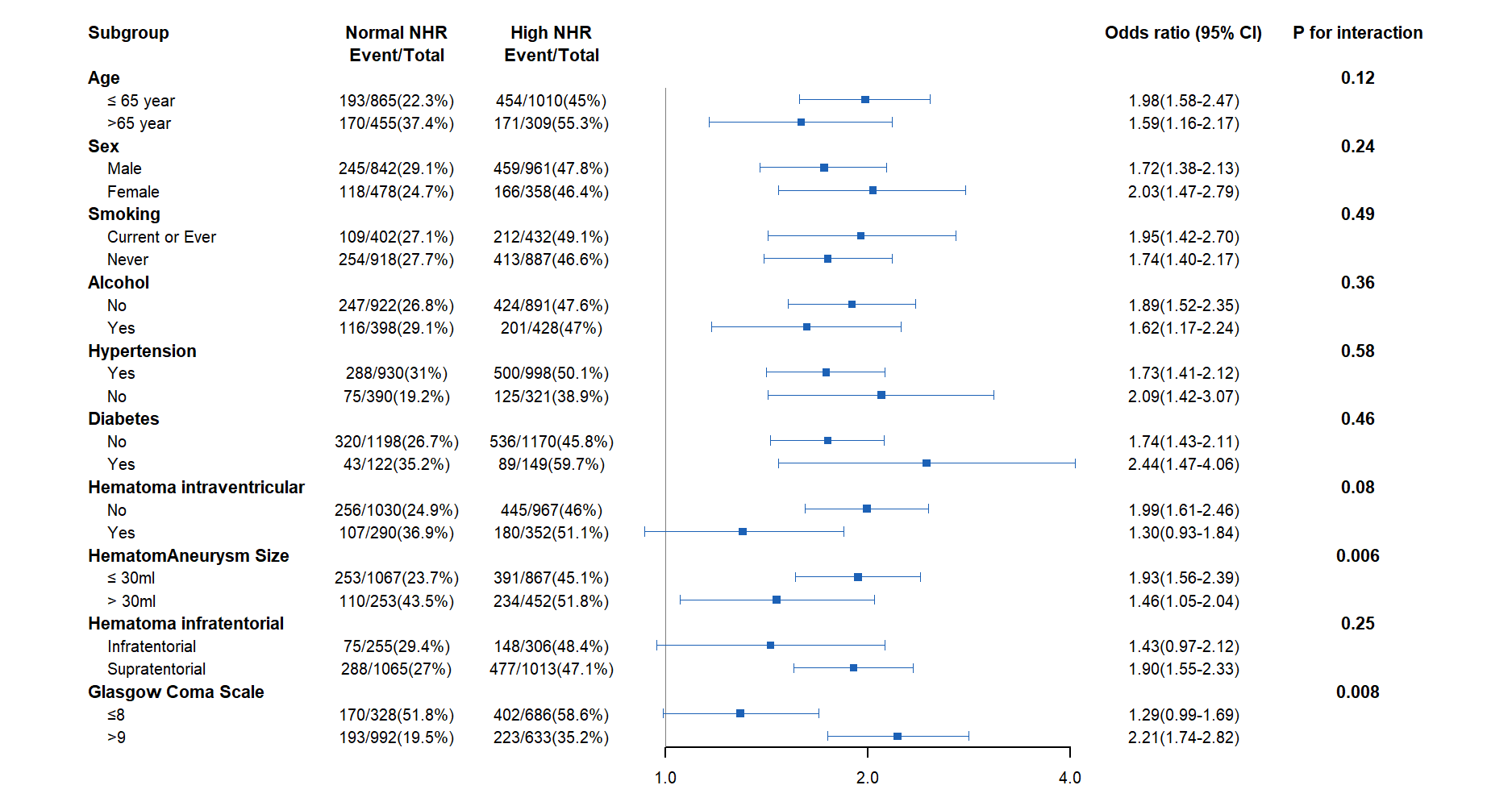

Supplement: Supplementary file 2 [file Image_2.jpeg]

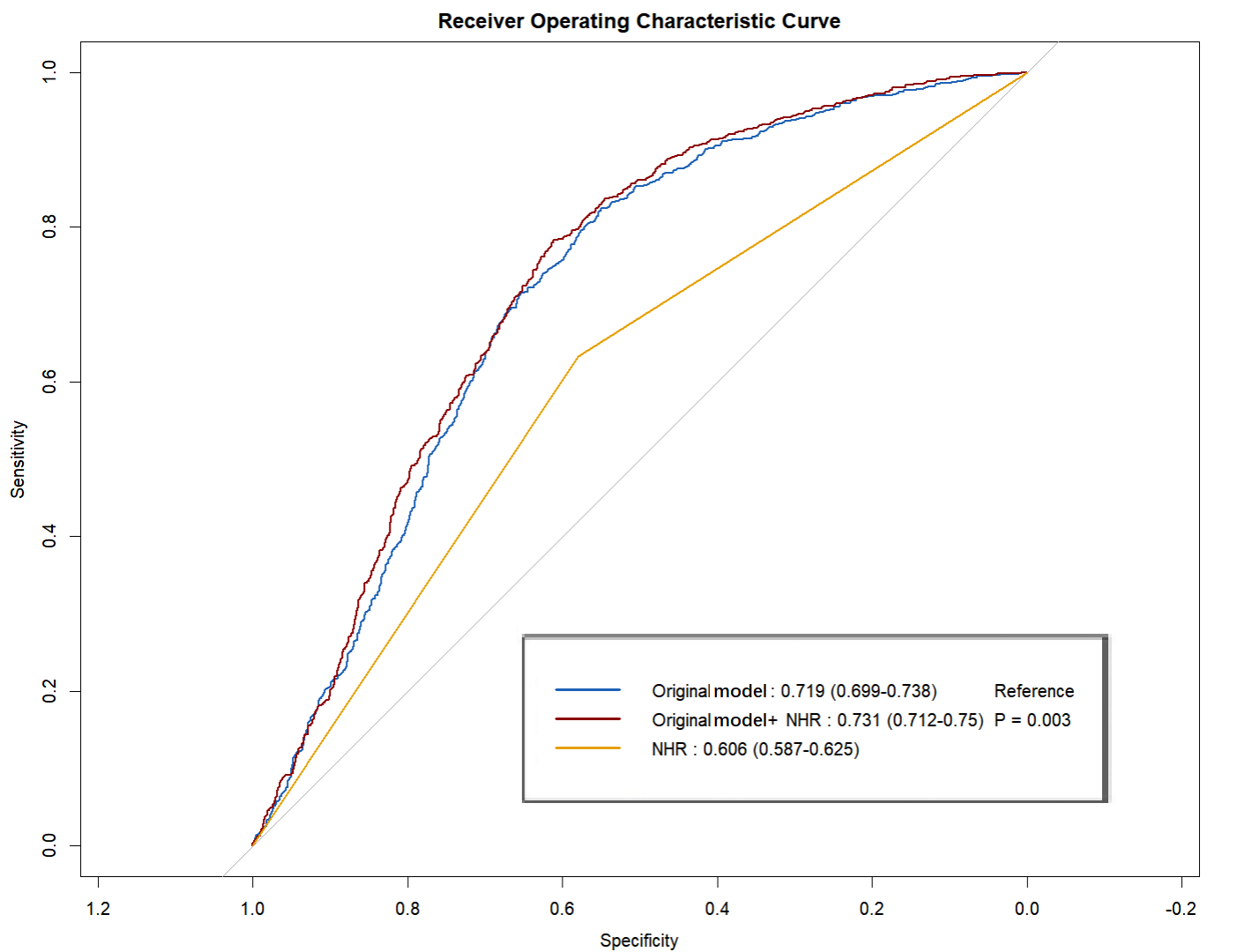

Supplement: Supplementary file 3 [file Image_3.jpeg]
